# Predictive values of the postoperative neutrophil-to-lymphocyte ratio, platelet-to-lymphocyte ratio, and lymphocyte-to-monocyte ratio for the diagnosis of early periprosthetic joint infections: a preliminary study

**DOI:** 10.1186/s13018-020-02107-5

**Published:** 2020-11-30

**Authors:** Guanglei Zhao, Jie Chen, Jin Wang, Siqun Wang, Jun Xia, Yibing Wei, Jianguo Wu, Gangyong Huang, Feiyan Chen, Jingsheng Shi, Jinyang Lyu, Changquan Liu, Xin Huang

**Affiliations:** Department of Orthopedics, Huashan Hospital, Fudan University, No.12 Middle Urumqi Road, Shanghai, 200040 China

**Keywords:** NLR, PLR, LMR, Total joint arthroplasty, Periprosthetic joint infection

## Abstract

**Background:**

Several studies have been conducted to report diagnostic values of the neutrophil-to-lymphocyte ratio (NLR), platelet-to-lymphocyte ratio (PLR), and lymphocyte-to-monocyte ratio (LMR) in the many diseases, such as oncological, inflammatory, and some infectious diseases. However, the predictive value of these laboratory parameters for early periprosthetic joint infections (PJIs) has not yet been reported. The aim of this study was to determine predictive values of the postoperative NLR, PLR, and LMR for the diagnosis of PJIs.

**Methods:**

In this retrospective study, 104 patients (26 early PJI cases and 78 non-PJI cases) who underwent total joint arthroplasty were enrolled in this study. All the patients were then categorized into two groups: PJI group, patients with the diagnosis of PJI (26 patients; 14 males, 12 females; mean age = 65.47 ± 10.23 age range = 51–81 ) and non-PJI group, patients without PJI (78 patients; 40 males, 38 females; mean age = 62.15 ± 9.33, age range = 41–92). We defined “suspected time” as the time that any abnormal symptoms or signs occurred, including fever, local swelling, or redness around the surgical site between 2 and 4 weeks after surgery and before the diagnosis. Suspected time and laboratory parameters, including NLR, PLR, LMR, erythrocyte sedimentation rate (ESR), and C-reactive protein (CRP), were compared between both groups. The trends of postoperative NLR, LMR, PLR, CRP, and ESR were also reviewed. The predictive ability of these parameters at the suspected time for early PJI was evaluated by multivariate analysis and receiver operating characteristic (ROC) curve analysis.

**Results:**

NLR, PLR, and LMR returned to preoperative levels within 2 weeks after surgery in the two groups. In the PJI group, NLR and PLR were significantly increased during the incubation period of infection or infection, and LMR was significantly reduced, although 61.5% (16/26) of the patients had normal white blood cells. Interestingly, ESR and CRP were still relatively high 2 weeks after surgery and were not different between the two groups before infection started (*p* = 0.12 and 0.4, respectively). NLR and PLR were significantly correlated with early PJI (Odds ratios for NLR and PLR = 88.36 and 1.12, respectively; *p* values for NLR and PLR = 0.005 and 0.01, respectively). NLR had great predictive ability for the diagnosis of early PJI, with a cut-off value of 2.77 (sensitivity = 84.6%, specificity = 89.7%, 95% CI = 0.86–0.97).

**Conclusions:**

ESR and CRP seem not to be sensitive for the diagnosis of early PJI due to their persistently high levels after arthroplasty. The postoperative NLR at the suspected time may have a great ability to predict early PJI.

## Background

Total joint arthroplasty (TJA), including total knee arthroplasty (TKA) and total hip arthroplasty (THA), has become the most effective method for end-stage OA. However, the early diagnosis of periprosthetic joint infection (PJI), one of the most disastrous complications of TJA, is still a challenging task for surgeons [1]. Early PJI, defined as PJI within 4 weeks after index arthroplasty, has been used by many studies [2, 3]. It is much more difficult to diagnose early postoperative PJI than chronic PJI, which can be assisted by infectious traits such as pain, redness, inflammatory exudation and sinus. The International Consensus on PJI recommends C-reactive protein(CRP) > 100 mg/L, synovial WBC count > 10,000 cells/mL, and % polymorphonuclear neutrophils of 90% as diagnostic cut-offs for early postoperative PJI (< 6 weeks from index surgery). However, due to the long duration of the return of ESR to normal levels after surgery (usually at least 3–8 weeks), it is not sensitive to early postoperative PJI. CRP levels return to preoperative levels within 3 weeks; however, it may be elevated by various causes in addition to postoperative infection because elderly patients with comorbid illnesses frequently undergo TJA [4, 5].

Recently, the neutrophil-to-lymphocyte ratio (NLR), platelet-to-lymphocyte ratio (PLR), and lymphocyte-to-monocyte ratio (LMR) have been studied and found to be valuable in predicting the outcomes or prognosis of many diseases, such as oncological diseases, inflammatory diseases and some infectious diseases [6–10]. Moreover, Gölge UH [11] found NLR can be used as marker for PJI together with the other markers as ESR and CRP to increase the accuracy of the diagnosis by comparing preoperative and postoperative 6th month NLR values of the patients. They found that NLR may be valuable in predicting chronic PJI. As far as we know, however, the predictive value of these parameters in serum for early PJI has not been reported.

The aims of this study are (1) to examine the trends of NLR, PLR, and LMR after TJA and (2) to determine predictive values of these parameters for the diagnosis of early PJIs.

## Methods

**Fig 1.**
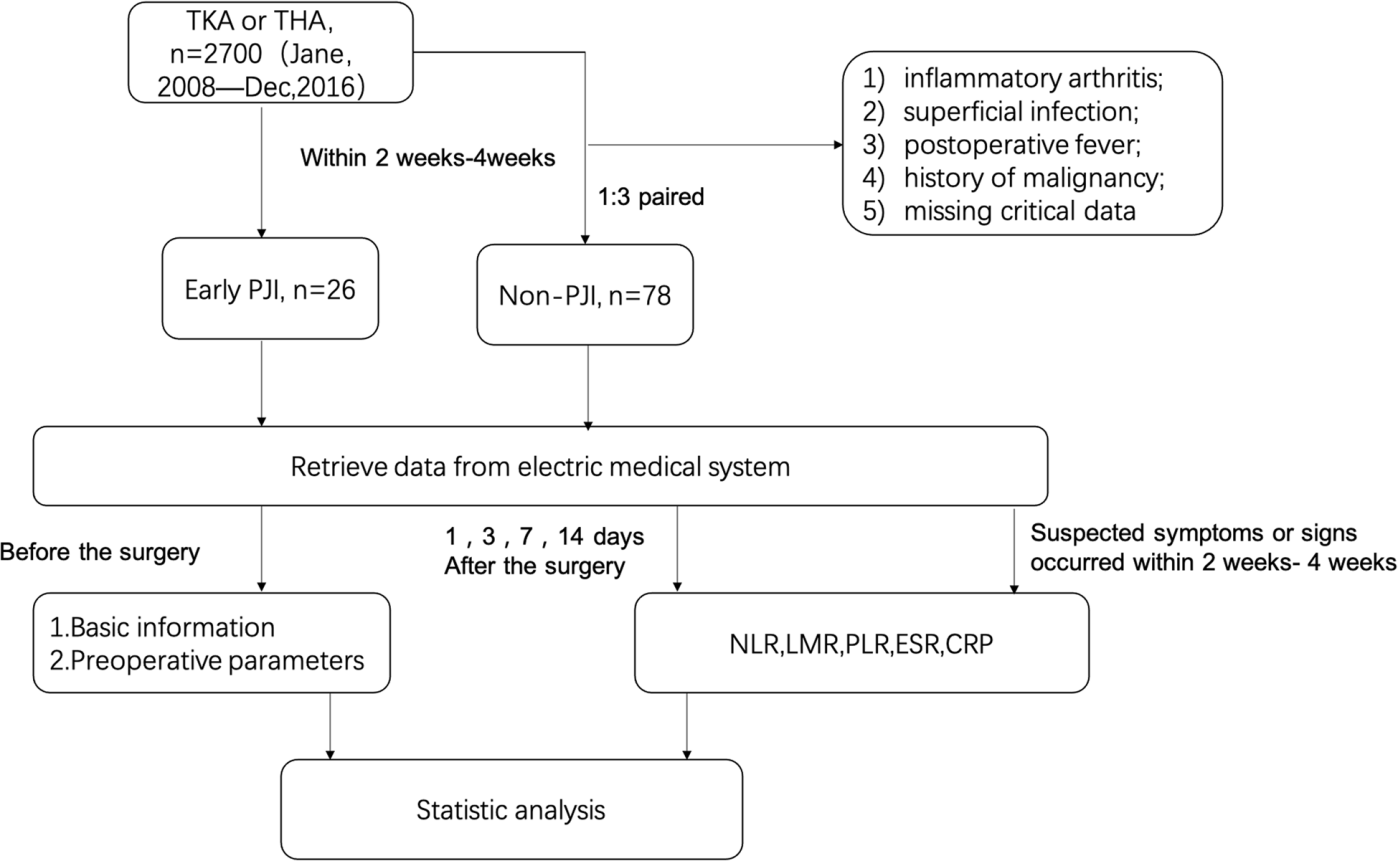
Flow diagram for study

The study protocol was approved by the Institutional Review Board of our Hospital, and informed consent was obtained from all patients. The Hospital Follow-up System (HFS, an electronic database comprising complete medical records for inpatient and outpatient patients) was used in our study(Fig. 1). We reviewed the patients diagnosed with early PJI (within 4 weeks after surgery) from February 2008 to December 2016 according to the criteria recently proposed by the Musculoskeletal Infection Society [12]. Patients with inflammatory arthritis, such as rheumatoid arthritis, ankylosing, and spondylitis were excluded in order to ruling out interference with other possible preconditions associated with elevated inflammatory markers. Also, excluded were (1) patients with superficial infection; (2) postoperative fever; (3) a history of malignancy; and (4) missing critical data. In total, 30 patients diagnosed with early PJI from February 2008 to December 2016 in our department were enrolled in this study. To make the results more reliable, we excluded PJI cases that occurred within 2 weeks after surgery and only included PJI cases that occurred between weeks 2 and 4. We defined “suspected time” as any abnormal symptoms or signs that occurred, including fever, local swelling, or redness around the surgical site between 2 and 4 weeks after surgery and before the diagnosis was made. It is routine to observe laboratory tests at 1 and 3 days after surgery in our department during the hospital stay of the patient. ESR, CRP, and routine blood examination were requested at 7 and 14 days when the patients left the hospital and went to a community hospital, and the data were uploaded by apps (WeChat). Patients’ basic information (including age, sex, height, weight, and body mass index (BMI)) and the results of preoperative laboratory tests (including neutrophil (N), lymphocyte (L), monocyte (M), and platelet (P) counts, ESR, and CRP) were obtained. Finally, 26 patients were included in the final analyses. A control group was matched at a ratio of 1:3 by sex, BMI, year of surgery, and Charlson-Deyo scores. The Charlson-Deyo value is a weighted score derived from the sum of the scores for each of the comorbid conditions listed in the Charlson comorbidity score [13]. All the included data were compared between the two groups. Moreover, the postoperative levels of ESR, CRP, neutrophil-to-lymphocyte ratio (NLR), lymphocyte-to-monocyte ratio (LMR), and platelet-to-lymphocyte ratio (PLR) were observed in the two groups after the operation. Pearson correlation analysis was conducted to evaluate the relationships between early PJI and the parameters at the suspected time. Receiver operating characteristic (ROC) analysis and multivariate analysis were also performed to determine the predictive value of these hematological parameters at the suspected time for early PJI.

### Statistical analysis

All the statistics are presented as the mean ± SD for continuous variables and the median (P25, P75) for discrete variables. Clinical characteristics were compared between the PJI group and the non-PJI group. The continuous variables of different groups were compared by using Student’s *t* test or the Mann–Whitney *U* test, and categorical variables were tested using Pearson’s *χ*^2^ test. In ROC analysis, the optimal cut-off values of several markers, including NLR, PLR, and LMR, that best predict the possibility of early PJI were determined with the maximum value of Youden’s index, which was calculated by sensitivity + specificity-1. All statistical analyses were performed with SPSS 22.0 (SPSS Inc., Chicago, IL, USA). All tests were two-sided, and statistical significance was defined as when the *p* value was less than 0.05.

## Results

A total of 104 patients (PJI group: *N* = 26; non-PJI group: *N* = 78) were included in this study. As shown in Table 1, the mean age was 65.47 ± 10.23 years in the PJI group and 62.15 ± 9.33 years in the non-PJI group. The preoperative NLR and PLR in the PJI group were higher than those in the non-PJI group, and the preoperative LMR was slightly lower in the PJI group. However, there was no significant difference in any of the preoperative parameters, including age, BMI, Charlson-Deyo score, WBC, neutrophil count, lymphocyte count, platelet count, NLR, PLR, LMR, ESR, and CRP, between the two groups.

**Table 1:** Patient basic characteristics

	PJI group (***N*** = 26)	Non-PJI group (***N*** = 78)	***t***/*χ*^**2**^	***P*** value
**Age (years)**	65.47 ± 10.23	62.15 ± 9.33	1.53	0.13
**Male (% total)**	14 (53.8%)	40 (51.3%)	0.39	0.53
**BMI (kg/m**^**2**^**)**	24.15 ± 2.37	23.32 ± 2.75	1.38	0.17
**White blood cell (WBC × 10**^**9**^**)**	6.32 ± 1.66	6.61 ± 2.12	0.63	0.53
**Neutrophil count (NE × 10**^**9**^**)**	3.75 ± 1.18	3.84 ± 2.08	0.21	0.83
**Lymphocyte count (LM × 10**^**9**^**)**	1.72 ± 0.57	1.82 ± 0.63	0.71	0.48
**Monocyte counts (MO × 10**^**9**^**)**	0.45 ± 0.20	0.46 ± 0.35	0.14	0.89
**Platelet count**	220.83 ± 56.96	214.18 ± 61.69	0.48	0.63
**ESR (mm/h)**	18.13 ± 14.88	15.62 ± 12.96	0.82	0.41
**CRP (mg/L)**	9.27 ± 15.41	7.10 ± 10.93	0.79	0.43
**NLR**	2.17 ± 0.40	2.11 ± 0.43	0.42	0.68
**PLR**	129.10 ± 51.68	114.42 ± 35.83	1.34	0.18
**LMR**	3.88 ± 1.60	3.96 ± 1.59	0.22	0.82

*PJI* periprosthetic joint infection, *BMI* body mass index, *ESR* erythrocyte sedimentation rate, *CRP* C-reactive protein, *NLR* neutrophil-to-lymphocyte ratio, *PLR* platelet-lymphocyte ratio, *LMR* lymphocyte-monocyte ratio

**Fig 2.**
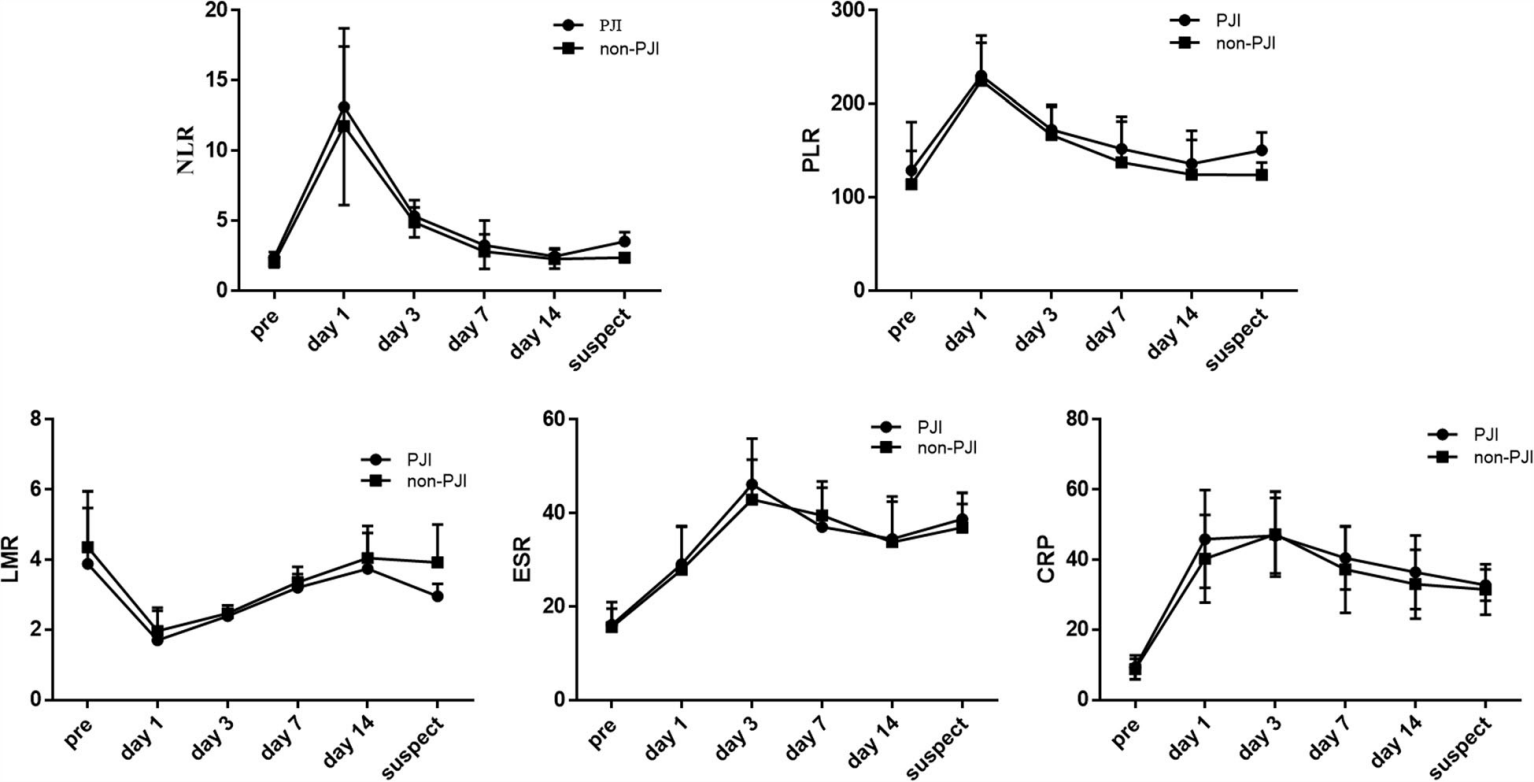
The overall trend of NLR, PLR, LMR, ESR, and CRP in the non-PJI and PJI group after the operation

The trends of postoperative NLR, PLR, LMR, CRP, and ESR at different time points in both groups are shown in Fig. 2. In the two groups, the above indexes except LMR increased rapidly on the first day and reached a peak 1–3 days after the operation, while LMR decreased significantly and then elevated gradually. In addition, NLR, PLR, and LMR returned to their preoperative levels within approximately 2 weeks. However, ESR and CRP remained higher (ESR > 20 mm/h, CRP > 20) 2 weeks after the operation. For the PJI group, the average suspected time point was 21.6 days; NLR, PLR, and LMR were significantly different compared with those in the non-PJI group (*p* < 0.01, Table 2), even with 61.5% (16/26) of patients with normal white blood cell levels at the suspected time.

**Table 2:** The parameters at the suspected time point

Variable	PJI group	Non-PJI group	***t***	***P*** value
**ESR**	38.80 ± 5.60	36.96 ± 5.04	1.57	0.12
**CRP**	32.77 ± 4.45	31.51 ± 7.19	0.84	0.4
**NLR**	3.52 ± 0.67	2.38 ± 0.38	10.76	< 0.01
**LMR**	2.96 ± 0.36	3.93 ± 1.09	4.45	< 0.01
**PLR**	150.69 ± 19.35	124.11 ± 13.34	7.8	< 0.01

In the association analysis (Table 3), the Pearson correlation coefficients for WBC, NLR, PLR, and LMR at the suspected time were obtained (*r*_WBC_ = 0.22, *r*_NLR_ = 0.72, *r*_PLR_ = 0.61, *r*_LMR_ = 0.39). However, there was no correlation between early PJI and the other parameters. The results of multivariate analysis suggested that the increased NLR and PLR are independent predictive indexes for early PJI (Table 3, OR_NLR_ = 88.36, OR_PLR_ = 1.12, *P*_NLR_ = 0.005, *P*_PLR_ = 0.01).

**Table 3:** Pearson correlation relevant analysis and multivariate analysis between the PJI and the parameters at the suspected time

	Pearson correlation		Multivariate analysis		
Variable	***r***	***P*** value	OR	95% CI	***P*** value
**White blood cell (WBC × 10**^**9**^**)**	0.22	0.02*	0.98	0.64–1.50	0.93
**Neutrophil count (NE × 10**^**9**^**)**	0.20	0.04*	1.12	0.81–1.54	0.49
**Age (years)**	0.17	0.09	1.04	0.93–1.17	0.46
**BMI (kg/m**^**2**^**)**	0.14	0.15	1.02	0.67–1.55	0.93
**ESR (mm/h)**	0.15	0.12	1.08	0.89–1.32	0.42
**CRP (mg/L)**	0.08	0.41	0.98	0.81–1.19	0.81
**NLR**	0.72	< 0.01*	88.36	3.89–2004.61	0.005*
**PLR**	0.61	< 0.01*	1.12	1.03–1.23	0.01*
**LMR**	0.39	< 0.01*	0.41	0.09–1.83	0.24

*r* Pearson correlation coefficient, *OR* odds ratio, *CI* confidence interval, * means statistical difference, *BMI* body mass index, *ESR* erythrocyte sedimentation rate, *CRP* C-reactive protein, *NLR* neutrophil-to-lymphocyte ratio, *PLR* platelet-lymphocyte ratio, *LMR* lymphocyte-monocyte ratio

**Fig 3.**
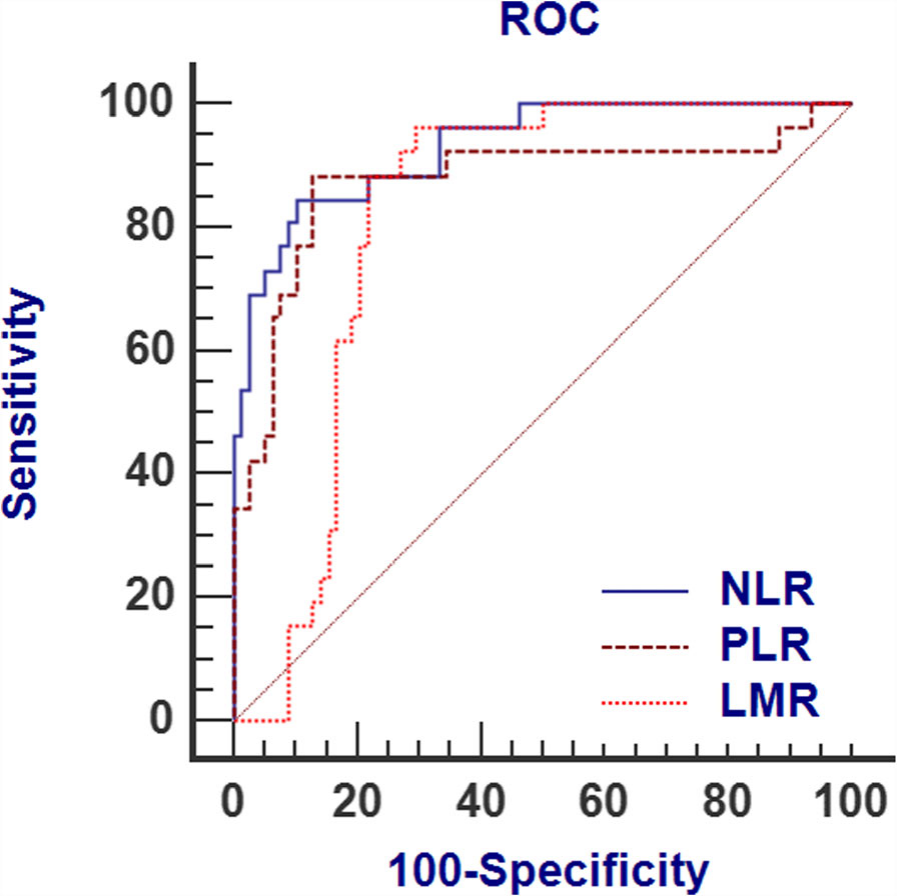
ROC curve of the NLR, PLR, and LMR at the suspected time

The ROC curves of NLR, LMR, and PLR at the suspected time are shown in Fig. 3. The results shown in Table 4 indicate that the areas under the ROC curve (AUCs) were all larger than the reference value (0.5). NLR had the highest area under the ROC curve (AUC) (AUC_NRL_ = 0.93, AUC_PLR_ = 0.87, AUC_LMR_ = 0.81). The cut-off value of NLR was 2.77 (sensitivity = 84.6%, specificity = 89.7%, 95% CI = 0.86–0.97).

**Table 4:** AUC of the NLR, PLR, and LMR at suspected time

Variable	AUC	Cut-off	Sensitivity (%)	Specificity (%)	95% CI
**NLR**	0.93	2.77	84.6	89.7	0.86–0.97
**PLR**	0.87	139.22	88.5	87.2	0.79–0.93
**LMR**	0.81	3.40	88.5	78.2	0.72–0.88

*AUC* area under the curve, *NLR* neutrophil-to-lymphocyte ratio, *PLR* platelet-lymphocyte ratio, *LMR* lymphocyte-monocyte ratio

## Discussion

In our study, we found NLR at the suspected time is significantly higher in early PJI patients than non-infected controls and early PJI could be predicted high accuracy if NLR is > 2.77 at the suspected time defined in our study.

The traditional inflammatory markers (ESR and CRP) are highly effective for predicting PJI before revision arthroplasty [14]. However, they may not be suitable for early PJI [15]. In this study, we reviewed the trend of the postoperative CRP, ESR, NLR, LMR, and PLR levels within 2 weeks (Fig. 2). The persistent high level of ESR and CRP after surgery greatly limited their value in the screening or prediction of early PJI, which was consistent with the findings of published literature [4, 16]. However, we found NLR, LMR, and PLR returned to their preoperative levels within approximately 2 weeks, which means they are useful to screening early PJI. Other serum biomarkers, such as D-dimer [17, 18], interleukin-6 [19], tumor necrosis factor-α [20], procalcitonin (PCT), and α-defensin [21, 22], have been researched for diagnosing PJI, but these parameters are not easily accessible in some remote areas, and larger-scale studies are still needed to validate their effectiveness.

Among the various kinds of tests, it is very critical to find a simple and practical marker for diagnosing early PJI. NLR, PLR, and LMR can be easily obtained by routine blood tests. They have been demonstrated as stable and cost-effective biomarkers that reflect the inflammatory response as they mediate inflammation by various biochemical mechanisms, such as release of arachidonic acid metabolites and platelet-aggravating factors. Hiroyuki [23] found NLR at 3–4 and 6–7 days postoperatively were useful markers for the early prediction of SSI in patients who had undergone spinal decompression surgery. These parameters may aid in identifying patients at higher risk of SSI after spinal decompression surgery. Moreover, Yombi, J. C et al. [16] found that NLR has a faster normalization than CRP. It is potentially a better biomarker to follow post-operative inflammation or early infection after TKA. However, they only focus on the post-operative inflammation and did not study PJI. In our study, the results showed that NLR and PLR at the suspected time are independent factors associated with early PJI. According to the ROC analysis, NLR may be more valuable than PLR, and the cut-off value for predicting early PJI was 2.77, which is similar with the result of Gölge UH’s research [11]. This means that early PJI could be predicted high accuracy if NLR is > 2.77 at the suspected time defined in our study. Gölge UH’s research focus on the value of NLR in predicting chronic PJI; however, NLR seems to be very sensitive to inflammatory stimulation, which may be more suitable for the diagnosis of acute PJI.

There are several limitations in our study. First, the major drawback of our study is that we did not include data on any probable effect of antibiotic use or the type of pathogens found in PJI cultures on the ratios. Second, the findings of the study, even if sound, would not be generalizable to PJI within 2 weeks or after 4 weeks postoperatively. Third, the sample size in our study was relatively small, and this was a retrospective study. Fourth, only the perioperative period (within 7 days) and the first follow-up point (2 weeks) were analyzed in patients without early PJI because postoperative follow-up is routinely conducted at 2 weeks, 3 months, and 6 months after discharge in our department. In addition, NLR and PLR also increased significantly due to the surgery and returned to the preoperative level within 2 weeks, so we chose patients diagnosed with PJI 2 weeks after the operation. Moreover, whether there is a difference in the threshold value of NLR between TKA and THA could not be obtained from the present study. Therefore, larger-scale, prospective studies, and subgroup analyses are needed to further investigate the ability of NLR to predict early PJI.

## Conclusion

In conclusion, we found that the rise and fall of NLR, PLR, and LMR is more rapid than serum CRP and ESR postoperatively. What is more, NLR can be considered a useful tool for the diagnosis and clinical monitoring of early PJI at the suspected time after total joint arthroplasty. Further validation work is still required to reproduce these findings and confirm the relative test performance of NLR versus other more established serum markers.

## Data Availability

The raw data will be made available from the authors upon reasonable request.

## Abbreviations

NLR: Neutrophil-to-lymphocyte ratio
PLR: Platelet-to-lymphocyte ratio
LMR: Lymphocyte-to-monocyte ratio
PJI: Periprosthetic joint infection
ESR: Erythrocyte sedimentation rate
CRP: C-reactive protein
ROC: Receiver operating characteristic
OR: Odds ratio
OA: Osteoarthritis
TJA: Total joint arthroplasty
TKA: Total knee arthroplasty
THA: Total hip arthroplasty
HFS: Hospital Follow-up System
BMI: Body mass index
ROC: Receiver operating characteristic

## References

[1] Zimmerli W, Trampuz A, Ochsner PE (2004). Prosthetic-joint infections. N Engl J Med.

[2] Bryan AJ, Abdel MP, Sanders TL, Fitzgerald SF, Hanssen AD, Berry DJ (2017). Irrigation and debridement with component retention for acute infection after hip arthroplasty: improved results with contemporary management. J Bone Joint Surg Am.

[3] Tsukayama DT, Goldberg VM, Kyle R (2003). Diagnosis and management of infection after total knee arthroplasty. J Bone Joint Surg Am.

[4] Windisch C, Brodt S, Roehner E, Matziolis G (2017). C-reactive protein course during the first 5 days after total knee arthroplasty cannot predict early prosthetic joint infection. Arch Orthop Trauma Surg.

[5] Kim TW, Kim DH, Oh WS, Sim JA, Lee YS, Lee BK (2016). Analysis of the causes of elevated C-reactive protein level in the early postoperative period after primary total knee arthroplasty. J Arthroplast.

[6] Kahramanca S, Ozgehan G, Seker D, Gokce EI, Seker G, Tunc G (2014). Neutrophil-to-lymphocyte ratio as a predictor of acute appendicitis. Ulus Travma Acil Cerrahi Derg.

[7] Templeton AJ, McNamara MG, Seruga B, Vera-Badillo FE, Aneja P, Ocana A (2014). Prognostic role of neutrophil-to-lymphocyte ratio in solid tumors: a systematic review and meta-analysis. J Natl Cancer Inst.

[8] Vatankhah N, Jahangiri Y, Landry GJ, McLafferty RB, Alkayed NJ, Moneta GL (2017). Predictive value of neutrophil-to-lymphocyte ratio in diabetic wound healing. J Vasc Surg.

[9] Hsu JT, Wang CC, Le PH, Chen TH, Kuo CJ, Lin CJ (2016). Lymphocyte-to-monocyte ratios predict gastric cancer surgical outcomes. J Surg Res.

[10] Xia WK, Liu ZL, Shen D, Lin QF, Su J, Mao WD (2016). Prognostic performance of pre-treatment NLR and PLR in patients suffering from osteosarcoma. World J Surg Oncol.

[11] Gölge UH, Kilinc S, Kaymaz B, Pazarci O, Öztemur Z, Bulut O (2016). Neutrophil to lymphocyte ratio may be a diagnostic marker for prosthetic joint infection. J Clin Analytical Med.

[12] Parvizi J, Zmistowski B, Berbari EF, Bauer TW, Springer BD, Della VCJ (2011). New definition for periprosthetic joint infection: from the workgroup of the musculoskeletal infection society. Clin Orthop Relat Res.

[13] Austin SR, Wong YN, Uzzo RG, Beck JR, Egleston BL (2015). Why summary comorbidity measures such as the Charlson comorbidity index and elixhauser score work. Med Care.

[14] Parvizi J, Ghanem E, Menashe S, Barrack RL, Bauer TW (2006). Periprosthetic infection: what are the diagnostic challenges?. J Bone Joint Surg Am.

[15] Parvizi J, Gehrke T (2014). International consensus group on Periprosthetic joint I: definition of periprosthetic joint infection. J Arthroplast.

[16] Yombi JC, Schwab PE, Thienpont E (2016). Neutrophil-to-lymphocyte ratio (NLR) distribution shows a better kinetic pattern than C-reactive protein distribution for the follow-up of early inflammation after total knee arthroplasty. Knee Surg Sports Traumatol Arthroscopy.

[17] Shahi A, Kheir MM, Tarabichi M, Hosseinzadeh HRS, Tan TL, Parvizi J (2017). Serum D-dimer test is promising for the diagnosis of periprosthetic joint infection and timing of reimplantation. J Bone Joint Surg Am.

[18] Xu H, Xie J, Huang Q, Lei Y, Zhang S, Pei F (2019). Plasma fibrin degradation product and d-dimer are of limited value for diagnosing periprosthetic joint infection. J Arthroplast.

[19] Randau TM, Friedrich MJ, Wimmer MD, Reichert B, Kuberra D, Stoffel-Wagner B (2014). Interleukin-6 in serum and in synovial fluid enhances the differentiation between periprosthetic joint infection and aseptic loosening. PLoS One.

[20] Bottner F, Wegner A, Winkelmann W, Becker K, Erren M, Gotze C (2007). Interleukin-6, procalcitonin and TNF-alpha: markers of peri-prosthetic infection following total joint replacement. J Bone Joint Surg Br.

[21] Xie K, Qu X, Yan M (2017). Procalcitonin and alpha-defensin for diagnosis of periprosthetic joint infections. J Arthroplast.

[22] Li B, Chen F, Liu Y, Xu G (2017). Synovial fluid alpha-defensin as a biomarker for peri-prosthetic joint infection: a systematic review and meta-analysis. Surg Infect.

[23] Inose H, Kobayashi Y, Yuasa M, Hirai T, Yoshii T, Okawa A (2019). Procalcitonin and neutrophil lymphocyte ratio after spinal instrumentation surgery. Spine (Phila Pa 1976).

